# PD-L1 degradation pathway and immunotherapy for cancer

**DOI:** 10.1038/s41419-020-03140-2

**Published:** 2020-11-06

**Authors:** Qian Gou, Chen Dong, Huihui Xu, Bibimaryam Khan, Jianhua Jin, Qian Liu, Juanjuan Shi, Yongzhong Hou

**Affiliations:** 1 Department of Oncology, The Affiliated Wujin Hospital, Jiangsu University, Changzhou, Jiangsu Province 213017 China; 2 School of Medicine, Jiangsu University, Zhenjiang, Jiangsu Province 212013 China; 3 School of Life Sciences, Jiangsu University, Zhenjiang, Jiangsu Province 212013 China; 4 Department of Oncology, The Wujin Clinical College of Xuzhou Medical University, Xuzhou, Jiangsu Province 212017 China

**Keywords:** Immunosurveillance, Lysosomes, Ubiquitylation

## Abstract

Programmed death ligand 1 (PD-L1, CD274) is an essential immune checkpoint protein that binds to programmed death 1 (PD-1) on T-lymphocytes. T cell plays a critical role in killing cancer cells while the cancer cell exhibits immune escape by the expression of PD-L1. The binding of PD-L1 to PD-1 inhibits T cell proliferation and activity, leading to tumor immunosuppression. Increasing evidence shows that PD-L1 protein undergoes degradation in proteasomes or lysosomes by multiple pathways, leading to enhanced immunotherapy for cancer. Although some specific drugs induce PD-L1 degradation and increase antitumor activity, the combination of these drugs with PD-L1/PD-1 blockade significantly enhances cancer immunotherapy. In this review, we have discussed the interaction of PD-L1 degradation with cancer immunotherapy.

## Facts

1. PD-L1 is an essential immune checkpoint protein that binds to PD-1 on T cells, which plays a critical role in killing cancer cells, while cancer cell exhibits immune escape by the expression of PD-L1.
2. Increasing evidence shows that PD-L1 protein will be degraded in proteasomes or lysosomes, leading to enhanced immunotherapy for cancer.
3. Some specific drugs or a combination of these drugs with PD-L1/PD-1 blockade inhibitors can effectively enhance antitumor immunotherapy.

## Open questions

1. How does GSK3β or AMPK induce the extracellular fragment of PD-L1 phosphorylation?
2. It remains unclear that how membrane PD-L1 protein can be translocated into the cytoplasm and degraded. Is there any other E3 ligase or autophagy receptor for PD-L1 degradation by proteasomes or lysosomes?
3. Does the FDA-approved agents that target PD-L1 (atezolizumab, etc.) or PD-1 (nivolumab, etc.) induce PD-L1 degradation?
4. Although some specific drugs or a combination of these drugs with PD-L1/PD-1 blockade inhibitors can effectively enhance antitumor immunotherapy, the mechanism of PD-L1 degradation remains unclear.

## Introduction

**Fig. 1:**
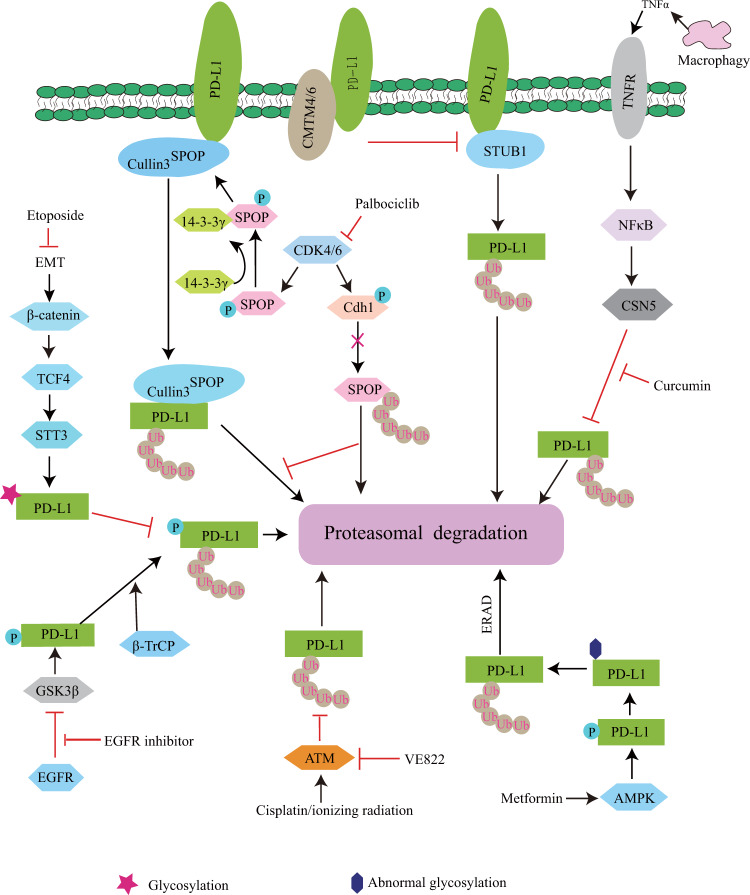
The pathways of PD-L1 ubiquitination and degradation. PD-L1 undergoes ubiquitination and degradation by E3 ubiquitin ligases, including STUB1, Cullin3^SPOP^, and β-TrCP, which is abolished by CMTM4/6, CSN5, and STT3. Although glycosylation of PD-L1 increases its protein stability, the AMPK agonist or EGFR inhibitor reverses this process and induces PD-L1 proteasome-dependent degradation. Moreover, in response to extracellular stimuli, PD-L1 protein triggers ubiquitination and degradation by multiple pathways.

**Fig. 2:**
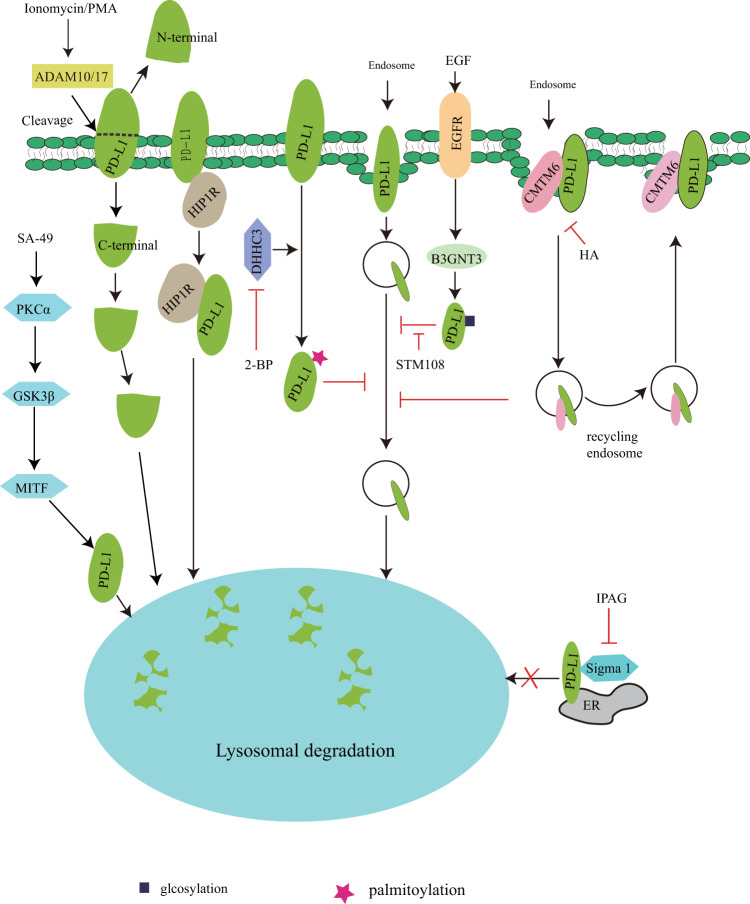
The pathways of PD-L1 autophagic degradation. HIP1R, PKCα/GSK3β/MITF, ADAM10/17, and endosomal sorting-signal induce PD-L1 protein degradation by autophagy, which is inhibited by CMTM6, DHHC3, and Sigma I. In response to extracellular stimuli or specific anti-PD-L1 antibody such as HA or STM108, PD-L1 protein is degraded via autophagy.

The host immune system exhibits the ability of antitumor activity by activation of the immune response^1,2^. As a “don’t find me” signal, the programmed death ligand 1 (PD-L1), a critical immune checkpoint protein, binds to programmed death 1 (PD-1) on T cells, leading to cancer immunosuppression^3^. The binding of PD-L1 to PD-1 on T cells results in the dephosphorylation of the T-cell receptor (SHP-1/2). It inhibits T cells from killing cancer cells by reducing T cell proliferation and activity^4^. In contrast, the immune checkpoint inhibitors such as PD-L1 or PD-1 monoclonal antibodies have been used for cancer treatment, including melanoma, non-small-cell lung cancer, gastric cancer, and breast cancer^5^. Although PD-1/PD-L1 blockade therapy exhibits significant clinical benefits for multiple types of cancer, the response rates of patients are less than 40% with an unclear mechanism^6^. The high expression of PD-L1 protein levels is observed in different types of cancers, which promotes cancer cell immune escape^5,7^. The expression of PD-L1 in cancer cells is regulated by multiple signaling pathways, including NFκB, MAPK, mTOR, STAT, and c-Myc^8,9^, while PD-L1 protein undergoes degradation in proteasomes or lysosomes by multiple pathways^10–16^, leading to increased effectiveness of cancer immunotherapy (Figs. 1 and 2 and Tables 1 and 2).

**Table 1:** PD-L1degradation and antitumor activity.

Degradation by	Regulatory signal	Therapy	Caner types	Reference
Proteasome	EGFR/GSK3β	Osimertinib	NSCLC	^33^
Proteasome	mTORC2/Akt/GSK3β	MTI-31	NSCLC	^34^
Proteasome	ATR	VE822	Breast cancer	^24^
Lysosome	PKCα/GSK3β/MITF	SA-49	NSCLC	^27^
Lysosome	Sigma 1	IPAG	Prostate cancer, TNBC	^32^
Lysosome	ZDHHC3	2-BP	Colon cancer	^29^
Lysosome	HIP1R	PD-LYSO	Colon cancer	^15^

**Table 2:** Combination therapy.

Degradation by	Regulatory signal	Therapy	Cancer types	Reference
Proteasome	EGFR/GSK3β/β-TrCP	Gefitinib + anti-PD-1	Colon cancer, TNBC	^13^
Proteasome	NFκB/CSN5	Curcumin + anti-CTLA4	TNBC, colon cancer, melanoma	^22^
Proteasome	AMPK	Metformin + CTLA4	Breast cancer, lung cancer	^21^
Proteasome	EMT/β-catenin/STT3	Etoposide + anti-Tim-3	Colon cancer, TNBC	^23^
Lysosome	EGFR/B3GNT3	STM108-MMAE conjugate	TNBC	^31^
Lysosome	CMTM6	H1A + cisplatin	Breast cancer, colon cancer	^30^
Proteasome	CDK4/6/ cullin3^SPOP^	Palbociclib + anti**-**PD-1	Colon cancer	^11^

## The pathways of PD-L1 ubiquitination and degradation

The ubiquitin-proteasome system plays an important role in the regulation of protein stability, which consists of ubiquitin-activating enzyme (E1), ubiquitin-conjugating enzyme (E2), and ubiquitin ligase (E3) that delivers ubiquitin from E2 to the specific substrates^17–19^. PD-L1 undergoes ubiquitination and degradation by E3 ubiquitin ligases such as STUB1^10^, Cullin3^SPOP^ ^11^, and β-TrCP (β-transducin repeat-containing protein)^12,13^. Although STUB1 ubiquitin ligase destabilizes PD-L1 protein by inducing its lysosomal degradation in A375 melanoma cells^10^, the mechanism is still unclear. In contrast, Zhang et al.^11^ described the detailed mechanism of PD-L1 ubiquitination and degradation by the cyclinD-CDK4/SPOP/Cdh1 pathway. Mechanistically, cyclinD-CDK4 mainly induced SPOP phosphorylation at serine-6, resulting in the recruitment of 14-3-3γ to SPOP and thereby inhibiting APC/Cdh1-mediated SPOP degradation; consequently, this promoted PD-L1 ubiquitination and degradation by SPOP ubiquitin ligase. However, SPOP function loss by mutations enhanced PD-L1 protein stability, resulting in tumor immunosuppression. Since glycogen synthase kinase 3β (GSK3β) can induce phosphorylation and degradation of multiple substrates by proteasomes^20^, the interaction of GSK3β with PD-L1 induces its phosphorylation at tyrosine-180/serine-184, resulting in β-TrCP ubiquitin ligase-mediated PD-L1 ubiquitination and degradation^13^. In addition, activation of AMP-activated protein kinase (AMPK) induces PD-L1 phosphorylation at serine-195, leading to abnormal PD-L1 glycosylation and ER-associated protein degradation (ERAD)^21^.

Although PD-L1 undergoes ubiquitination and degradation, cancer cells exhibit the ability to inhibit this process. Mezzadra et al.^10^ reported that the cellular membrane protein CMTM4/6 interacted with PD-L1, leading to inhibition of PD-L1 ubiquitination and degradation, which consequently impaired T cell activity. In the tumor microenvironment, macrophage-secreted TNFα activates NFκB in cancer cells, leading to increased deubiquitinase CSN5 (COP9 signalosome 5) gene transcription and expression, and CSN5 stabilizes PD-L1 protein by inhibiting its ubiquitination and degradation, resulting in cancer cell immune escape^22^. In response to EGF, active EGFR induces GSK3β phosphorylation, leading to inhibition of the binding of GSK3β to PD-L1, and facilitates PD-L1 glycosylation; consequently, this inhibits PD-L1 degradation by β-TrCP ubiquitin ligase^13^. Since PD-L1 glycosylation enhances PD-L1 protein stability^13^, epithelial-mesenchymal transition (EMT) triggers β-catenin-induced STT3 (*N*-glycosyltransferase) gene transcription and expression, resulting in PD-L1 glycosylation, which subsequently inhibits PD-L1 degradation in cancer stem cells^23^. In response to cisplatin or ionizing radiation, activated ATM (Ataxia-telangiectasia) increases PD-L1 protein stability by inhibiting its proteasome-dependent degradation in MDA-MB-231 cells resulting in reduced T cell activity^24^, whereas the mechanism of PD-L1 degradation is unclear. This finding suggests that chemotherapy or radiation could decrease the response rates of PD-L1/PD-1 blockade by increasing PD-L1 expression in cancer cells. Taken together, PD-L1 undergoes ubiquitination and degradation, while cancer cell exhibits the ability to inhibit this process by multiple pathways resulting in tumor immunosuppression (Fig. 1).

## The pathways of PD-L1 degradation by autophagy

Autophagy induces degradation of cytoplasmic materials and organelles in lysosomes, which plays an important role in maintaining cellular homeostasis^25,26^. In addition to the proteasome-dependent degradation discussed above, PD-L1 undergoes autophagic degradation by HIP1R and PKCα/GSK3β/MITF pathways^15,16^. HIP1R contains a lysosomal targeted signal and binds to PD-L1, which subsequently delivers PD-L1 into lysosomes for autophagic degradation and enhances T cell killing of cancer cells^15^. In addition to the directly regulatory mechanism of HIP1R-mediated PD-L1 autophagic degradation, SA-49 activates PKCα/GSK3β/MITF pathway-mediated lysosome biogenesis, leading to PD-L1 autophagic degradation; consequently this enhances T cell activity and inhibits tumor growth^27^. Since autophagy is usually non-selective degradation of substrates^25^, why does increased lysosome biogenesis degrade only PD-L1 protein rather than other intracellular proteins? This needs to be further addressed. Romero et al.^16^ reported that the region (225–240 aa) of PD-L1 was the potential surface metalloproteases (ADAM10/17) cleavage site in triple-negative breast cancer, which subsequently generated N-terminal (~24 kDa) fragments that were released outside and C-terminal (~13 kDa) fragments that were degraded by lysosomes, and the activators of ADAM10/17 (ionomycin/PMA) enhanced this event, whereas the mechanism of PD-L1 degradation by lysosomes is still unclear.

Although HIP1R induces PD-L1 autophagic degradation^15^, cancer cells have exhibited the ability to inhibit PD-L1 autophagic degradation by binding to CMTM6 or palmitoylation modification by DHHC3 (palmitoyltransferase ZDHHC3)^28,29^. The binding of CMTM6 to plasma membrane PD-L1 and recycling endosomes, leading to inhibition of endocytosed PD-L1 degradation, subsequently enhances PD-L1 protein stability and promotes tumor immune escape^28^, whereas H1A (PD-L1 antibody) abolishes the binding of PD-L1 to CMTM6, resulting in PD-L1 degradation by lysosomes^30^. PD-L1 modification by glycosylation and palmitoylation results in inhibition of its endosomal sorting-mediated autophagic degradation^29,31^. In response to EGF, active EGFR induces *N*-glycosyltransferase B3GNT3 expression, leading to B3GNT3-mediated glycosylation of PD-L1, which subsequently inhibits PD-L1 degradation resulting in immunosuppression in a breast xenograft tumor model^31^. Palmitoyltransferase DHHC3 induces PD-L1 palmitoylation at cystine-272, inhibits its ubiquitination and endosomal sorting-mediated autophagic degradation, and subsequently enhances PD-L1 protein stability and immune suppression in a colon tumor model^29^. On the other hand, Sigma 1 mainly binds to glycosylated PD-L1 and maintains PD-L1 protein stability. In contrast, Sigma 1 inhibitor IPAG induces PD-L1 autophagic degradation in breast and prostate cancer cells, thereby leading to enhanced T cell activity^32^. Collectively, PD-L1 undergoes autophagic degradation, whereas cancer cells exhibit the ability to maintain its protein stability, leading to tumor immunosuppression (Fig. 2).

## PD-L1 degradation and antitumor activity

Cancer cells exhibit the ability to inhibit PD-L1 degradation and maintain its protein stability by deubiquitination or glycosylation of PD-L1^13,22,23^, while PD-L1 induces proteasome-dependent degradation by the GSK3β pathway in response to osimertinib or MTI-31 in EGFR mutant non-small cell lung cancer (NSCLC) cells^33,34^, and MTI-31 induces PD-L1 degradation and increases T-cell proliferation, which is associated with inhibition of tumor growth in a lung cancer tumor model^34^. Moreover, the ATR kinase inhibitor VE822 induces proteasomal degradation of PD-L1, leading to increased T cell killing of breast cancer cells^24^. In addition to proteasomal degradation, SA-49-induced PD-L1 autophagic degradation by the PKCα/GSK3β/MITF pathway results in enhanced T cell killing of cancer cells^27^. Similarly, Sigma 1 inhibitor IPAG induces PD-L1 autophagic degradation in breast and prostate cancer cells, leading to increased T cell activity^32^. Pharmacological inhibition of palmitoyltransferase DHHC3 by 2-bromopalmitate (2-BP) promotes PD-L1 autophagic degradation and enhances antitumor activity in a colon tumor model^29^. In addition, the chimeric PD-LYSO peptide with PD-L1 binding and lysosomal sorting sequences of HIP1R effectively targets PD-L1 for autophagic degradation and increases T cell killing of colon cancer cells^15^. These findings suggest that PD-L1 degradation by treatment with drugs effectively enhances tumor immunotherapy (Table 1).

## Combination therapy

Since PD-L1 protein undergoes degradation in cancer cells in response to the drugs gefitinib^13^, curcumin^22^, metformin^21^, and etoposide^23^ when combined with anti-PD-1, anti-CTLA4, or anti-Tim3 antibody, we observe that combination therapy effectively improves tumor immunotherapy (Table 2). The specific anti-glycosylated PD-L1 (gPD-L1) antibody could target glycosylated PD-L1, resulting in PD-L1 degradation; thus, the conjugated STM108 (anti-gPD-L1) with MMAE (monomethyl auristatin E) effectively enhances antitumor activity in a breast tumor model^31^. In addition, the combination of H1A, a specific anti-PD-L1 antibody for PD-L1 autophagic degradation, with cisplatin significantly increases antitumor activity^30^. Either CDK4/6 or mTOR inhibitors increase PD-L1 protein levels by disruption of CDK4/6/cullin3^SPOP^ or mTORC1/p70S6K/β-TrCP pathway-mediated PD-L1 ubiquitination and degradation^11,12^, while the combination of CDK4/6 inhibitors with PD-L1/PD-1 blockade effectively enhances tumor immunotherapy^11^. These findings suggest that the effect of antitumor drugs could be counteracted by increasing PD-L1 expression, leading to cancer cell immune escape. Still, the combination of inhibitors with PD-1/PD-L1 blockade may provide a strategy for cancer therapy. Taken together, the rational combination therapy could effectively enhance antitumor activity (Table 2).

## Conclusion

Increasing evidence suggests that PD-L1 protein degradation effectively promotes cancer immunotherapy (Table 1) and the combination therapy significantly enhances this event (Table 2), which provides a potential strategy to increase the response rates of PD-1/PD-L1 blockade in cancer immunotherapy. Although PD-L1 antibody (H1A, STM108) could induce PD-L1 degradation in lysosomes^30,31^, it is still unclear whether the FDA-approved agents that target PD-L1 (atezolizumab, etc.) or PD-1 (nivolumab, etc.) could induce PD-L1 degradation. The mechanism of PD-L1 degradation is elusive in some studies such as the interaction of CMTM6 with PD-L1 leading to inhibition of PD-L1 degradation by both ubiquitination^10^ and autophagy^30^, and hence it is needed to further determine the correlation of these two pathways. In addition, inhibition of the mTOR pathway reduces PD-L1 protein levels in NSCLC cell lines^34,35^, but the other reports are opposite in the same type of cancer cells^12^. These contradictory findings may be derived from different PD-L1 antibodies or inhibitors. GSK3β/β-TrCP or AMPK/ERAD pathway induces PD-L1 ubiquitination and degradation. As a secreted trans-membrane protein, although PD-L1 protein is synthesised in the cytoplasm, it will be targeted to the endoplasmic reticulum (ER) by its signal peptide and enter into the ER. How does GSK3β or AMPK induce the extracellular fragment of PD-L1 phosphorylation? Moreover, it remains unclear how membrane PD-L1 protein can be translocated into the cytoplasm and degraded. Is there any other E3 ligase or autophagy receptor for PD-L1 degradation by proteasomes or lysosomes? Furthermore, does the cleaved cytoplasm fragment of PD-L1 by ADAM10/17^16^ have an additional intracellular function? These issues need to be further clarified, which may contribute to the understanding of cancer immunosuppression by PD-L1/PD-1 blockade for cancer patients.
